# Trends in the removal of Aboriginal children in Western Australia.

**DOI:** 10.23889/ijpds.v7i3.1972

**Published:** 2022-08-25

**Authors:** Passiona Cottee, Ximena Camacho, Natalie Cooper, Katie Irvine

**Affiliations:** 1 TD School, University of Technology, Sydney; 2 Centre for Big Data Research in Health, Faculty of Medicine, UNSW Sydney; 3 Australian Institue of Health and Welfare; 4 NSW Department of Premier and Cabinet, Sydney, Australia

## Objectives

People with disability represent 1 in 6 Australians and use services provided across multiple levels of government. Data is siloed between different governments and service systems, limiting visibility and ability to improve outcomes. Our objective was to progress people-centred data sharing across jurisdictions at scale, focused on people with disability.

## Approach

More than 140 engagements with the disability community, governments, researchers, and service providers were conducted to ascertain user and data needs; systematically map enablers and barriers to data use; and outline the requisite technical capabilities and social license for a disability asset. Five demonstration projects tested novel linkage approaches, data flows and access models to inform technical aspects of the design. We adopted an iterative try-test-learn approach and forged consensus through extensive dialogue across all stakeholders to collaboratively design a system for enduring data linkage. The final design was reviewed by external experts to ensure best practice and build trust.

## Results

A national disability-focused data asset requires linking data across all human services portfolios, as will be required for most national assets seeking to improve outcomes for particular communities or in particular domains. The proposed approach is three building blocks: national data linkage and integration infrastructure, national data system governance, and streamlined data sharing agreements for multiple uses. Current project-by-project approaches to linkage are proposed to be replaced with new enduring national keys to connect disparate state-based infrastructure and enable streamlined, reusable data curation processes. Collaboration between jurisdictions will be embedded through joint governance of the new infrastructure. Finally, a new approach to data sharing agreements between Australian governments will be developed to create safe parameters for streamlined access within existing legislation and authorisation pathways.

## Conclusion

By designing to meet information needs across human services, this approach can be leveraged for multiple linked, longitudinal enduring data asset, beyond disability. It multiplies the benefits of upfront efforts to share and improve data, reflects a shared understanding among stakeholders, and moves Australia towards scaled use of people-centred data.

